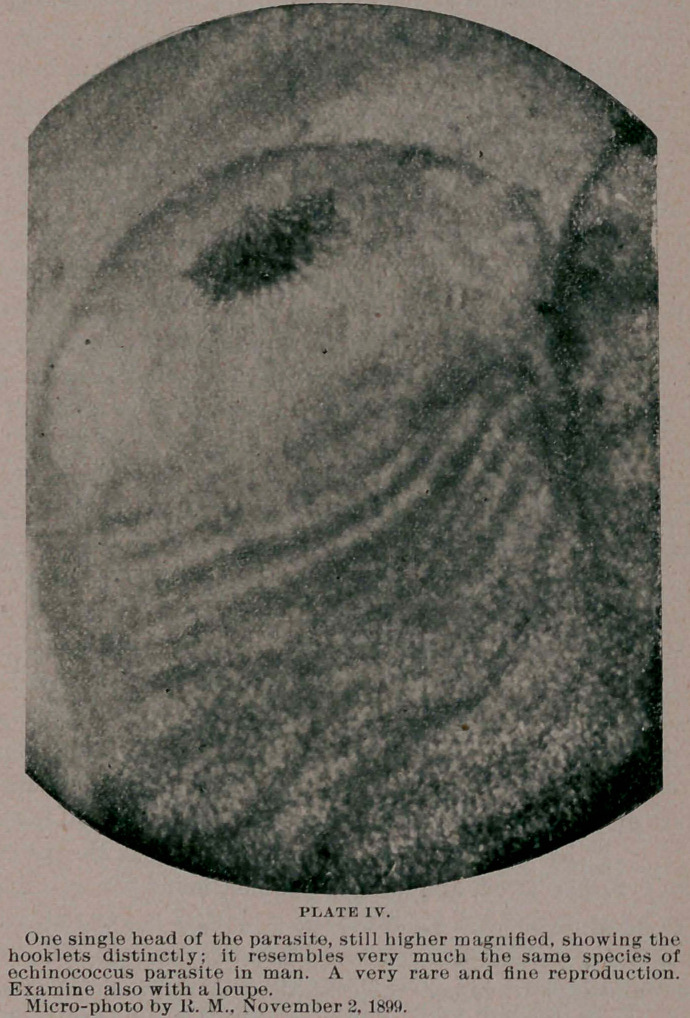# Some Observations on the Echinococcus Disease, Especially as Related to the Present Epidemic among the Prairie Rabbit

**Published:** 1899-11

**Authors:** R. Menger

**Affiliations:** San Antonio


THE
### TEXAS MEDICAL JOURNAL.

ESTABLISHED JULY, 1885.

PUBLISHED MONTHLY.—SUBSCRIPTION $1.00 A YEAR.

VOL. XV. AUSTIN, NOVEMBER, 1899. No. 5.

### Some Observations on the Echinococcus Disease, Especially as Related to the Present Epidemic Among the Prairie Rabbit.

BY R. MENGER, M. D., SAN ANTONIO.

*Editors Texas Medical Journal:*

(The near relationship of the echinococcus parasite of man to the same parasite disease in animal, and the question often asked of the writer whether this disease was the cause of the widespread and common tapeworm pest, induced me to investigate this interesting matter thoroughly in the following paper. There is no doubt to me as to others who have given the matter a close consideration, that there occur many cystic tumors in man which are really echinococci in advanced and degenerated state, but were overlooked as such for want of a microscopic examination. My paper, therefore, and the photo-micrographs of the echinococcus parasites, I hope, will be welcomed in throwing a little more light on the matter. —R. Menger.):

The scarcity of any detailed literature on some particular species of parasite disease in certain animals and especially the prevalence of an epidemic of a peculiar cystic disease in the prairie rabbit, the so-called *echinococcus parasite,* leads me to a few reflections, which, I hope will be interesting enough to call your attention to same for a few moments.

To hunters it is well known that among the rabbits it is nearly exclusively the large so-called jack rabbit which is often infested with peculiar tumorlike protuberances or cysts in different parts of its body and that, especially during a protracted dry season, a perfect epidemic of the disease exists among the large prairie rabbit, and that the small bush rabbit is nearly exempt from the disease.

In consulting a number of works, medical as well as veterinary, I find no mention of the echinococcus disease among the rabbits. It is a noticeable fact that, whilst this parasite in other animals invades nearly all the internal organs, the echinococcus tumors in rabbits, with but a few exceptional instances, have been found with particular preference in the muscular system, especially in the muscles of the lumbar region and the muscles of the thighs and ribs. A common idea prevails among the laity that these tumors are some sort of "veneral disease," "ulcerations," "grubworm," etc. I had occasion to examine a great number of cystic diseased rabbits, some of which, in rare instances, showed over one-half of the entire body covered with cysts, reaching along and between the muscles of the lumbar and thoracic region down to the abdominal and thoracic cavities from whence they generally start to develop and multiply or migrate to other parts of the body.

During their growth the cyst sacs protrude below the skin and they can easily be felt in the shape of roundish, oval or flat, single or conglomerated, and esaltic tumors, from the size of a marble up to a child's head, thereby giving the different parts of the rabbits' anatomy very deformed appearance. On dissecting these tumors, in advanced cases, a firm fibro-cystic membrane with the inner or endocyst, similar to the pyogenic membrane of some abscess cavities, is noticed, which is filled up with some albuminous like or gelatinous fluid, and each separate cyst, and even the surrounding base of such, contain myriads of small, whitish, hard kernels not larger than a pin's head. These kernels, if compressed between two slide glasses and examined with a magnifying glass, show an oval or roundish wormlike body with a short and rather broad neck, and a minute head. If put under microscopic examination, it is at once apparent that these bodies are some species of the larval state of the taenia echinococcus,—showing the peculiar segmented apartments across the body and neck of the worm, and the head-part shows a number of roundish sucking cups, generally four or eight, and a number of delicate curved hooklets. Some of the micro-photographs herewith submitted and prepared from a rabbit killed only a few days ago, show the histological appearance of these minute taenia bodies plainly. In general these echinococci of rabbit correspond to the echinococcus parasite of man, only that in man, whilst it is found in nearly all organs, but especially in the liver, it is a parasite that, as stated, nearly exclusively infests the muscular system of the rabbit—similar to the trichina spiralis of man, and some species of cysticerci—only that the latter in the rabbit, like the echinococcus of man, more often invade the liver, kidneys, diaphragm, lungs, pleura, intestines, the bulbus, pelvic organs (especially the sub-rectal tissues), lymphatics and sub-cutaneous cellular tissue,

PLATE I.

*Transverse section lumbar muscle of rabbit*, showing *echinococcus cysts* (1, 2, 3) imbedded in the muscle substance (4), with numerous scolices. *Cyst 1* is intact, with larval or embryonic tapeworms inside the delicate cyst membrane. *Cyst 2* shows a cyst burst, with numerous larval cystic tapeworms evacuated (5). *Cyst 3* is a younger cyst, also containing the embryonic tapeworm and germs.

Enlarged four times. Photo by R. M.

etc. The echinococcus tapeworm itself is described as a very small taenia, with only four or five joints.

The most accepted theory regarding the migration of the echinococcus into the substance of the liver in man is this one: that after the echinococcus embryo is set free in the intestine from the food or drink containing the ova, it starts on its migration into the portal vein, and through that source into the liver. Besides the portal vein and its hepatic branches, some authors claim the gall ducts and the lymphatic sinuses as the main source of migration into the liver substance, remnants of the parasites having been found within the lumen of those vessels The medium of transmission of the so-called *echinococcus multilocularis* or conglomerated cyst sacs has a somewhat different pathogeny than the other typical form, because well defined scoleces or parts of same are seldom encountered, and in Pepper's system of medicine on *echinococcus of the liver,* mention is made of an occasional anomalous development of the multilocular parasite, which, from its resemblance to colloid cancer, was supposed to have this character. Pepper states: "Its resemblance to colloid cancer is the more striking because of the tendency of the interior of the mass to undergo degeneration, to disintegrate, and to break up into pus sacs. An echinococcus multilocularis tumor is of almost stony hardness; it has a very dense fibrous structure, intersected by cavities with thick gelatinous cavities," etc. I have myself not encountered just such conditions as the above mentioned in examining echinococcus conglomerations in the rabbit, but I do recollect many years ago having operated a lady who had an enormous and nodulated cystic tumor of one of the ovaries, showing precisely the same conditions, but the case undoubtedly was one of cystoma or cystic degeneration of the ovarian pollicles; it weighed thirty pounds, the lady living yet, in good health. It is quite well known that in these slow growing cystomas, as well as in other more rapid growing neoplasms, strangulation and degeneration of the tumor substance with formation of pus cavities occur, even in small sized neoplasms.

In order to consider the prevalence and ethiology of the echinococcus disease, history shows that in India, where dogs are said to be very numerous, seventy per cent. of the cattle carry the echinococcus, and that in some other countries, Iceland for instance, according to Küchenmeister, the echinococcus disease is so widespread among man that the native physicians there in 1872 reported one-eighth of all the diseases occurring there as having been caused by the echinococcus parasite.

The propagation of the disease is thus described in Niemeyer's Pathology, in connection therewith: "Animals infested with the taenia echinococcus at times evacuate joints; the eggs or embryos contained therein, by some means get into the drinking water or come in contact with articles of food which are consumed raw. Having thus entered the alimentary canal, the minute embryos bore themselves with their six hooklets into the stomach walls or intestinal canal, until they gradually migrate further and eventually enter the liver or other organ. Here the small embryo swells up to a large cyst and in this cyst a colony of small unripe taenia or scoleces sprout up," etc.

The main cause of the disease in Iceland, according to Küchen-

PLATE II.

A few of the foregoing *embryonic tapeworms*, magnified about sixty times (the globular bodies were slightly compressed between two slide glasses). The black circular ring seen on each tænia head are the hooklets, and the white dots the sucking cups.

Micro-photo by R. M.

meister, is attributed to the many dogs kept there; these and the warm drinking water being responsible for the enormous spreading of the disease in man, as the dogs devour the cystic deposits which are carelessly thrown about the yards, and the people are reported to sleep with the dogs in one and the same hut in many instances. In the case of our jack rabbits, undoubtedly the same process of propagation or autoinfection takes place, and it is a noticeable fact that when our prairies are covered with an abundance of luxuriant green grass, cystic diseased rabbits are rarely met with; as soon though as a prolonged droughty season prevails, such as the present one, and the rabbits are compelled to eat nearly directly from the ground, they also undoubtedly devour a number of the echinococcus eggs or taenia embryos and the prairie is then found to be covered

PLATE III.

Four of the foregoing parasites higher magnified, showing two of the tænia heads with sucking cups and hooklets. In all of these parasites the neck part and entire body, as seen also on these micro-photos, is one cylindrical and ringed, curved, oval or roundish body, and the more developed heads of these parasites show numerous (as many as twelve) sucking cups.

Micro-photo by R. M., October, 1899.

with diseased rabbits. The wolves, and perhaps sheep also, undoubtedly spread the disease. The wolves kill and eat the remnants of the diseased or killed rabbits and deposit the ova or embryos in their manure.

It is an accepted fact by authorities (Leuckart, Siebold, Virchow, Küchenmeister and others) that the echinococcus is a sort of cystic tapeworm and the embryo state of the taenia echinococcus,—the same as the cysticercus cellulosæ is related to the taenia solium. Experimental tests with echinococci of man introduced into animals have proven negative so far, but, according to Niemeyer's pathology, it has been proven that animals fed on echinococci of another animal developed the taenia echinococci in the intestines of such animal experimented upon. The immense proliferating properties, each

PLATE IV.

One single head of the parasite, still higher magnified, showing the hooklets distinctly; it resembles very much the same species of echinococcus parasite in man. A very rare and fine reproduction. Examine also with a loupe.

Micro-photo by R. M., November 2, 1899.

vesicle containing, according to Friedberger (Pathology of the Domestic Animals), as many as thirty scoleces and in one echinococcus alone as many as a thousand; its very minute size and its vitality and tendency to multiply in the rabbit faster than the same species of parasite in man, readily explains the immense and widespread infection in the rabbit and canine species. Luckily though, we Texans are not living in Iceland, and our advanced civilized methods of preparing and cooking food, our protected and wholesome hydrant drinking water, and also the abandonment of eating raw meat, has cut a great figure and added immensely in the prophylaxis against all sort of parasite disease, and we owe it to our good housewives and hotel cooks in general that such parasitic disease as echinococcus *in man* is rather a very rare occurrence in Texas. Among Texas cattle and sheep this disease also is hardly known, as far as I am informed, and the question naturally arises: Is this parasite in our rabbit of the same species as the one that produces such havoc in some other countries? That, from its description and histological appearance, this embryo-coccus of the jack rabbit is near related to the echinococcus of man, there hardly can be any doubt. At any rate, though it has no relationship with the widespread and common tape-worm pest, the taenia medioconellata and solium, it is a parasitic disease sui generis as far as the Texas jack rabbit is concerned. The parasite, therefore, does not produce any tape-worm in man, as some persons are led to believe, and good cooking and frying will destroy the fins or cysts in the meat of any rabbit; but, of course, it is better that such infected rabbit meat should not be used at all.

In order to hear the opinion of other professional gentlemen on the subject I addressed, at this moment of writing, a few lines to Dr. F. Herff, now sojourning in Boerne, Texas (who by the way has had an immense amount of practical experience in this line also), and the venerable old gentleman kindly forwarded me the following reply on the subject: "The *taenia echinococcus* is a very small tape-worm with well developed head (scolex) and three or four joints (proglottides). It lives in the intestine of the dog and is not so very easy to find on account of its small size and the roundish appearance of the body, which at first sight looks like a small nematoid worm. Only by examining it in water, by which the intestinal mucus is washed away, and with a common loupe you can disclose its true organization. I stumbled on it during the examination I made on a dog which I had fed with trichinotic sausage, while hunting for intestinal trichinæ. Afterwards, I found it many times in the intestines of many dogs which were killed by the police during the rabies scare and furnished by Dr. Petterson, then city physician (in 1873). The embryonic state in the rabbit has been known to me long ago as it is also to many hunters who have shot rabbits and in consequence created a disgust in people to eat them. The embryo lives in the peritoneum and between the muscles in the connective tissues and is a true echinococcus, that is, a scolex, which multiplies in its cystic surrounding by sprouting or budding [see photo, Plate I], and creating new scolices. In that respect it differs from cysticercus which lives in a solitary cyst and to which family the taenia solium, mediocanellata, etc., belong, whilst the botriocephalus latus developed free in water at first, but probably enters then a host (cistern, sink or waterpool) and then is developed in the body of man as botriocephauls. The rabbit, of course, will not cause taenia in man, much less echinococcus, which is only produced by the ingestion of the eggs of the mature animal, the taenia echinococci which came from the dog that has eaten infected meat from rabbits, or through wolves or foxes who also harbor the mature parasite. In so far the eating of infected rabbit meat is only disgusting, but will not produce taeniæ The dog, however, is the evil doer and so the fondling and kissing of lap dogs or the sleeping in the same room with dogs is to be avoided. * * * I have found the echinococcus in man twice in the liver (in one case over two quarts and one quart in the other case); between the extensors of the thigh; one in the bulbus of an eye extirpated for panophthalmitis; once it passed from the bowels of a lady who had often suffered from pain in the liver, but did not show any tumor. My son took a great many echinococci from the bladder of a child; they were large, transparent and elongated. All these specimens I mentioned contained hooklets, and so the diagnosis was correct. I understand that Dr. McLaughlin, in Austin, also removed echinococci from the vagino-rectal cellular tissue. I am sure the disease is not so rare, and if proper inquiries were made it would prove to be so. The specimen of which you send the photograph is a true echinococcus as the multiplicity of the heads indicate."

## Figures and Tables

**PLATE I. f1:**
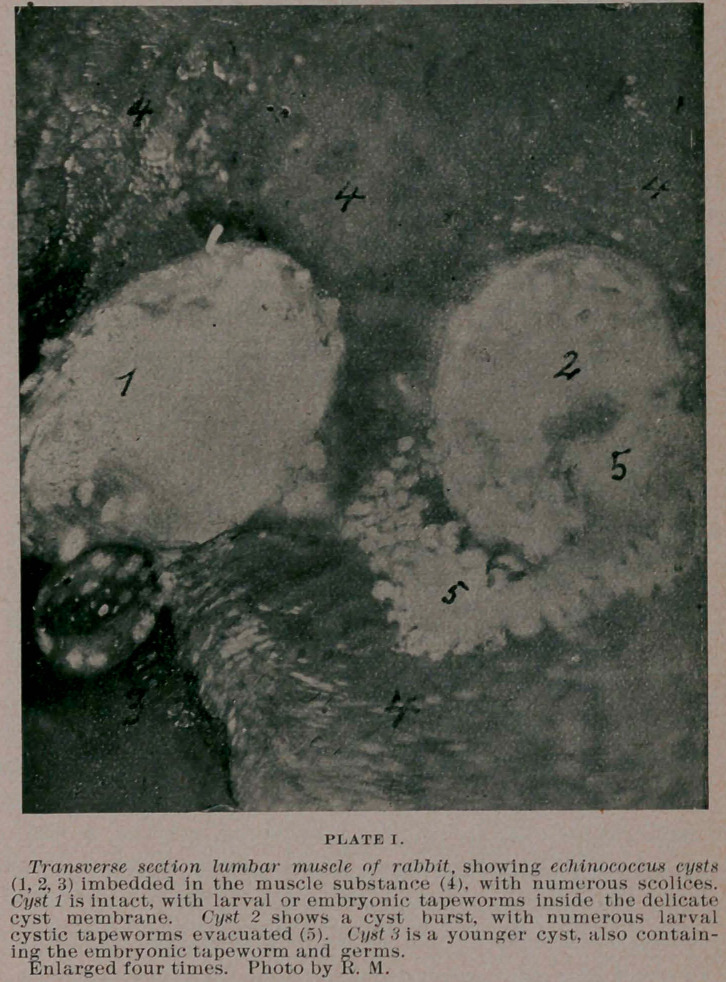


**PLATE II. f2:**
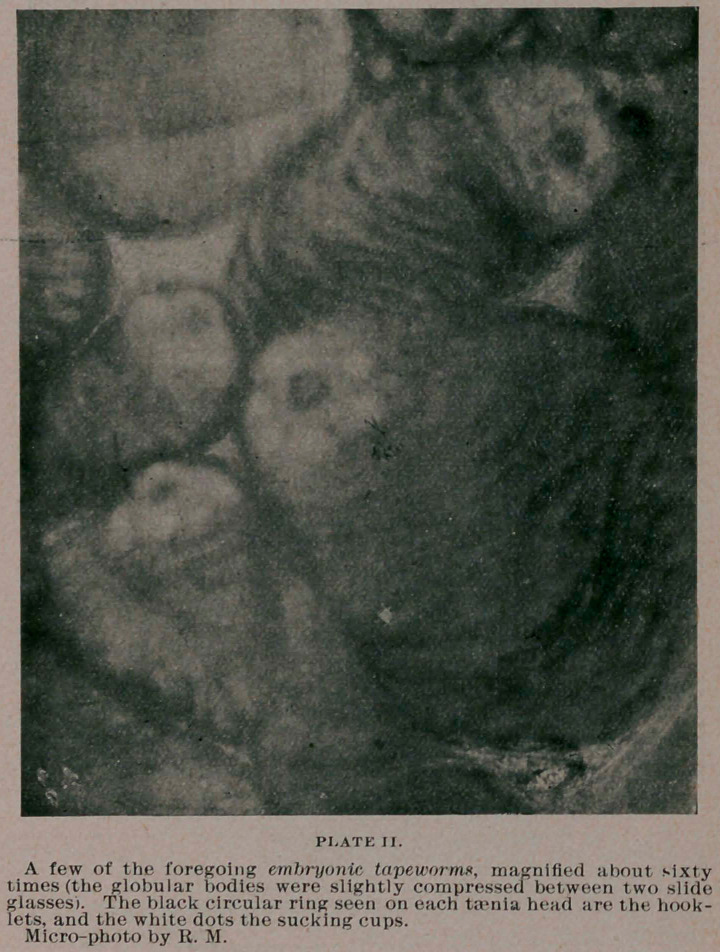


**PLATE III. f3:**
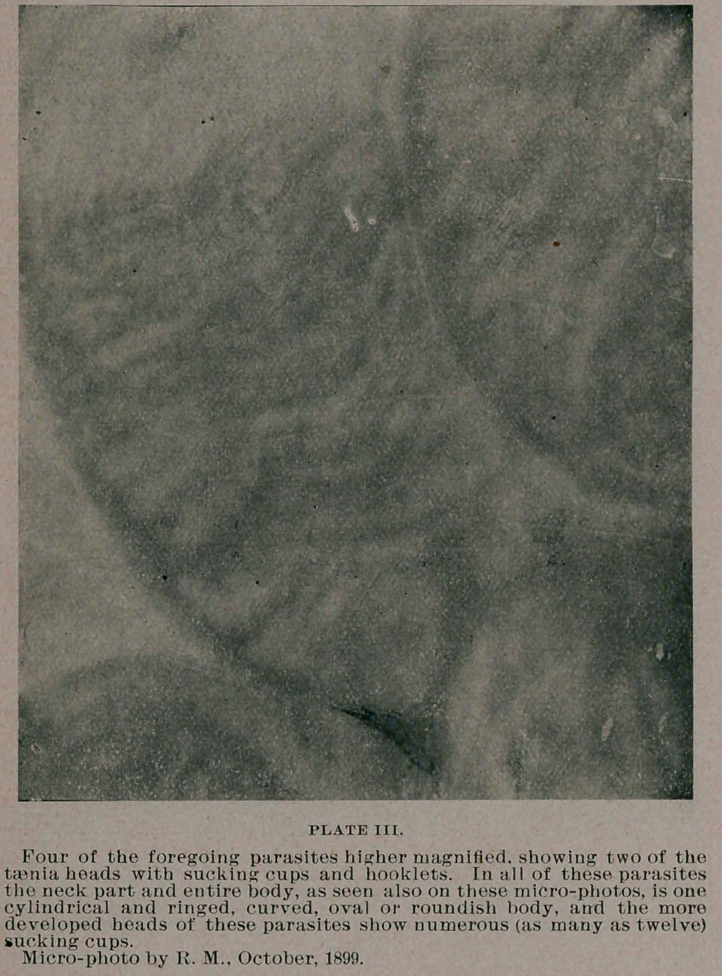


**PLATE IV. f4:**